# Deficiency of Interleukin-15 Confers Resistance to Obesity by Diminishing Inflammation and Enhancing the Thermogenic Function of Adipose Tissues

**DOI:** 10.1371/journal.pone.0162995

**Published:** 2016-09-29

**Authors:** Gregory Lacraz, Volatiana Rakotoarivelo, Sebastien M. Labbé, Mathieu Vernier, Christophe Noll, Marian Mayhue, Jana Stankova, Adel Schwertani, Guillaume Grenier, André Carpentier, Denis Richard, Gerardo Ferbeyre, Julie Fradette, Marek Rola-Pleszczynski, Alfredo Menendez, Marie-France Langlois, Subburaj Ilangumaran, Sheela Ramanathan

**Affiliations:** 1 Division of Immunology, Department of Pediatrics, Faculty of Medicine and Health Sciences, Université de Sherbrooke, CRCHUS, Sherbrooke, Québec, Canada; 2 Institut Universitaire de Cardiologie et de Pneumologie, Québec City, Québec, Canada; 3 Department of Biochemistry, University of Montréal, Montreal, Québec, Canada; 4 Department of Medicine, Faculty of Medicine and Health Sciences, Université de Sherbrooke, CRCHUS, Sherbrooke, Québec, Canada; 5 Department of Cardiology, McGill University, Montreal, Québec, Canada; 6 Department of Surgery, Faculty of Medicine and Health Sciences, Université de Sherbrooke, CRCHUS, Sherbrooke, Québec, Canada; 7 Department of Surgery, Laval University, CRCHU de Québec-Université Laval, Québec, Canada; 8 Department of Microbiology, Faculty of Medicine and Health Sciences, Université de Sherbrooke, CRCHUS, Sherbrooke, Québec, Canada; Universidade do Estado do Rio de Janeiro, BRAZIL

## Abstract

**Objective:**

IL-15 is an inflammatory cytokine secreted by many cell types. IL-15 is also produced during physical exercise by skeletal muscle and has been reported to reduce weight gain in mice. Contrarily, our findings on IL-15 knockout (KO) mice indicate that IL-15 promotes obesity. The aim of this study is to investigate the mechanisms underlying the pro-obesity role of IL-15 in adipose tissues.

**Methods:**

Control and IL-15 KO mice were maintained on high fat diet (HFD) or normal control diet. After 16 weeks, body weight, adipose tissue and skeletal mass, serum lipid levels and gene/protein expression in the adipose tissues were evaluated. The effect of IL-15 on thermogenesis and oxygen consumption was also studied in primary cultures of adipocytes differentiated from mouse preadipocyte and human stem cells.

**Results:**

Our results show that IL-15 deficiency prevents diet-induced weight gain and accumulation of lipids in visceral and subcutaneous white and brown adipose tissues. Gene expression analysis also revealed elevated expression of genes associated with adaptive thermogenesis in the brown and subcutaneous adipose tissues of IL-15 KO mice. Accordingly, oxygen consumption was increased in the brown adipocytes from IL-15 KO mice. In addition, IL-15 KO mice showed decreased expression of pro-inflammatory mediators in their adipose tissues.

**Conclusions:**

Absence of IL-15 results in decreased accumulation of fat in the white adipose tissues and increased lipid utilization via adaptive thermogenesis. IL-15 also promotes inflammation in adipose tissues that could sustain chronic inflammation leading to obesity-associated metabolic syndrome.

## Introduction

Chronic inflammation is an important mediator of obesity-associated metabolic syndrome [1–3]. Lipid deposition in white adipose tissues (WAT) stimulates resident macrophages to produce tumor necrosis factor α (TNFα), which stimulates adipocytes to secrete chemokines and thereby recruit immune cells [4, 5]. Thus, lipid-induced inflammatory mediators establish a vicious positive feedback loop and sustain chronic inflammation in obese WAT, leading to loss of insulin sensitivity and type 2 diabetes (T2D) [2]. In contrast to WAT, brown adipose tissue (BAT) helps to burn off fat (reviewed in [6]). BAT is characterized by abundant vascularization and adipocytes with high numbers of mitochondria and several small lipid droplets, giving a ‘multilocular’ appearance. Mitochondria present in BAT adipocytes express the ‘uncoupling protein 1’ (UCP1), which uncouples fatty acid oxidation from ATP generation, dissipating energy as heat, a process referred to as ‘adaptive, non-shivering thermogenesis’ [6]. Increase in brown-like adipocytes (brite or beige) in WAT correlates with lower susceptibility to obesity and diabetes [7]. In humans, BAT is present in newborns, but adults can also harbor significant deposits of UCP1-positive BAT in the supraclavicular and neck region [8–10]. While the role of inflammatory mediators in promoting insulin resistance in the obese WAT is well documented, it is not clear how inflammation affects the functions of BAT. TNFRα-deficient mice show increased thermogenesis due to deregulation in the central nervous system [11]. In contrast, catecholamines from alternatively activated macrophages promote adaptive thermogenesis [12]. Given that increasing the activity of BAT and induction of brite cells in WAT are considered to be a promising approach to decrease the fat mass in obese individuals [13–15], it is important to understand the role of inflammation in regulating adaptive thermogenesis.

The broad expression of IL-15 and its receptor (IL-15R) by multiple cell types and tissues, suggests a wide range of functions for IL-15 *in vivo*. IL-15, which is produced by macrophages in response to Toll-like receptor (TLR) ligands and IFNγ [16], is implicated in the generation, maintenance and activation of natural killer (NK) and CD8^+^ T-effector memory cells [17]. In addition, IL-15 has been reported to induce lipolytic activity in WAT, thereby decreasing lipid accumulation [18, 19]. These data, together with the high level of IL-15 expression in skeletal muscles following exercise, have led to the hypothesis that IL-15 may promote lean body mass [20]. Contrarily, other studies have reported increased expression of IL-15 in obesity and its induction by inflammatory cytokines in adipocytes [21]. Since IL-15 is induced by inflammatory stimuli, and several inflammatory cytokines such as IL-6, TNFα, IFNγ are implicated in promoting obesity and insulin resistance, we postulated that IL-15 might contribute to the pathogenesis of obesity rather than conferring protection against it. Indeed our findings on IL-15-deficient mice maintained on high-fat diet show that IL-15 is required for diet-induced weigh-gain, steatosis, insulin resistance and inflammation. Further analysis revealed that IL-15 compromises adaptive thermogenesis and promotes inflammation in adipose tissues.

## Materials and Methods

### Mice and experimental procedures

All animal procedures were carried out under protocols approved by the Ethics Committee for Animal Care and Use, Université de Sherbrooke. Mice were housed in micro-isolated sterile cages under specific pathogen-free conditions. *Il15*-deficient (*Il15*^*−/−*^) mice were purchased from Taconic (Germantown, USA) and backcrossed with C57BL/6 mice. *Il15*^*−/−*^ and (*Il15*^*+/+*^) mice were bred in our facilities. Apolipoprotein E-deficient (*Apoe*^***−/−***^***)*** mice in C57BL/6 background (Jackson Laboratories) were obtained from Dr. Pedro Juste D’Orleans (Université de Sherbrooke, Quebec, Canada). To induce obesity, 4-weeks-old animals were maintained for 16 weeks on high-fat diet (HFD) in which fat contributed to 60% of energy as Kcal (D12492l, Research Diets Inc., New Brunswick, NJ, USA). Control mice were fed normal chow diet (NCD). Mice had unlimited access to water and food. To evaluate resistance to hypothermia, animals were housed in individual cages at 10°C for 20h in a controlled environmental chamber with free access to food and water. Rectal temperature was determined using a pediatric rectal thermometer. During the duration of the experiments, none of the mice exhibited physical discomfort and none of them became ill. Mice were euthanized using a mixture of CO_2_ and O_2_ following isoflurane anesthesia at the end of experimental protocol. To stimulate the browning of adipose tissues, mice were treated with daily subcutaneous injections of CL-316243 (Sigma-Aldrich; USA; 1mg/kg body weight) or saline for 7 days.

### Human subjects and biopsy procedures

Adipose tissue-derived stem/stromal cells were extracted as described previously [22] from subcutaneous adipose tissue obtained by lipoaspiration or lipectomy from healthy female donors (36–46 years-old, body mass index (BMI) of 21.6–29.5) undergoing cosmetic surgery. Use of human samples was approved by the Human ethics committee at CRCHUS, Sherbrooke and Centre de Recherche du CHU de Québec-Université Laval. A written consent that was approved by the Human ethics committee was obtained from the participants.

### Uptake of non-esterified fatty acids (NEFAs) by BAT

Radiotracer experiments were performed under anesthesia [23]. Briefly, 13min prior the imaging experiment, mice received an intraperitoneal injection of CL-316243 (CL, 1.5mg/kg), or saline as control. Imaging experiments were performed with the Triumph^TM^ dual modality positron emission tomography coupled with computed tomography (PET/CT) scanner with 8cm axial FOV (Gamma Medica; Northridge, Canada) at the Sherbrooke Molecular Imaging Center. A bolus of radiopharmaceutical 14(R,S)-[(18)F]fluoro-6-thia-heptadecanoic acid (^18^FTHA), (17–20Mbq, in 0.2ml of 0.9% NaCl) was injected via the caudal vein over 60s. A 30-min dynamic acquisition was done to determine net NEFA uptake (*K*_*m*_) [23]. Dynamic data analysis of the images was performed as previously described [23].

### Indirect calorimetry

The Promethion High-Definition Room Calorimetry System was used for the indirect calorimetry studies (GA3, Sable Systems. Las Vegas, NV). Data acquisition and instrument control were coordinated by MetaScreen v. 1.6.2 and the obtained raw data was processed using ExpeData v. 1.4.3 (Sable Systems, Las Vegas, NV) using an analysis script detailing all aspects of data transformation. A standard 12h light/dark cycle (6:00–18:00) was maintained throughout the calorimetry studies. Prior to data collection, all animals were acclimated to cages for 4 days. Mice were subsequently placed in metabolic cages and data were acquired over 3 days.

### Glucose tolerance test and plasma lipid levels

Glucose tolerance test (GTT) and insulin tolerance test (ITT) were performed following standard methods [24]. For GTT, glucose (2g/kg body weight) was administered via intraperitoneal route. For ITT, insulin (1U/kg body weight) was administered via intravenous route. For both experiments, plasma glucose levels were measured using commercially available kits according to the manufacturer’s instructions (Cayman Chemical, USA). NEFA and cholesterol levels were evaluated using commercial kits (Cayman Chemical).

### Adipocytes cultures

Visceral WAT depots (epidydimal and retroperitoneal), subcutaneous, and BAT from 5 to 6-month old wild-type or *Il15*^−/−^ mice were removed, minced and digested with 1mg/ml collagenase type-I (Sigma-Aldrich) for 45min at 37°C in Dulbecco’s modified Earle’s medium (DMEM) (Sigma-Aldrich) containing 10% fetal bovine serum (FBS) and antibiotics. Digested tissues were filtered through 70μm nylon mesh and centrifuged at 325×g for 6min at 4°C. The floating fractions containing adipocytes were discarded and pellets representing the stromal vascular fraction (SVC) were resuspended and filtered through 40μm mesh. The cells were cultured in 12-well plates (5×10^5^ cells/well) at 37°C in DMEM-20% FBS. After reaching confluence, white or brown pre-adipocytes were differentiated into adipocytes following published methods [25]. In some experiments, the following agents were added to the differentiation media: recombinant IL-15 (10 or 20ng/ml), IFNγ (10ng/ml), TNFα (20ng/ml) or rosiglitazone (1μM). All cytokines for cell culture were obtained from R&D Systems (Minneapolis, USA).

Human adipose tissue-derived stromal cells [22] were seeded at 6.7–8.0×10^4^ cells/cm^2^ for expansion and cryopreserved after primary culture (passage 0). For experiments, cells were thawed, seeded at 5×10^5^ cells/well in a 12-well plate and cultured at 37°C for 4 days. Adipocyte differentiation was induced by treating confluent cultures of pre-adipocytes with 0.5mM isobutylmethylxanthine, 1μM dexamethasone, 850nM insulin, 0.2nM triiodo-L-thyronine (T3), and 0.1μM rosiglitazone for 72h. The cells were maintained for up to 21 days in medium containing 850nM insulin, 0.2nM T3, 1μM dexamethasone, and 0.1μM rosiglitazone [25]. To promote browning, cells were incubated with BMP7 (3nM) (Miltenyi Biotec; USA) for 48h before induction and also throughout the differentiation period. In some experiments, human recombinant IL-15 (10; 20ng/ml) or IFNγ (10ng/ml) was added 1h before adding BMP7.

### Measurement of the oxygen consumption rate (OCR)

The OCR was measured using the extracellular flux analyzer (XF96, Seahorse Bioscience; USA). After seeding equal amounts of stromal vascular cells (0.6×10^5^ cells per well), basal OCR was measured for 30min, followed by successive injections of oligomycin (1μM), FCCP (1μM), and rotenone + antimycin A (3μM each) using an instrument protocol of 2-min mix, 2-min wait, and 5-min measurement periods. Four measurements of OCR (in picomoles of O_2_ per minute) were recorded. Average values from 5–6 independent wells per experiment, normalized by DNA concentration, were calculated. In some experiments, the indicated cytokines were added during the last 18h of culture.

### Histology and immunohistochemistry (IHC)

Tissue samples fixed in buffered formalin and embedded in paraffin were sectioned and stained with hematoxylin and eosin (H&E). For IHC, sections were subjected to antigen retrieval by microwaving in 10mM citrate buffer (pH 6.0) for 15min, followed by blocking with 2% BSA in Tris-buffered saline. Endogenous peroxidase activity was inactivated with 0.03% H_2_O_2_ in water for 10min. Adipose tissue sections were stained overnight with UCP1 antibody (U6382, Sigma-Aldrich) at 4°C, washed, incubated with biotinylated secondary Ab (Jackson ImmunoResearch Laboratories, USA) and avidin-biotin complex (Vector Labs, USA), and visualized using diaminobenzidine (Sigma-Aldrich). For analyzing the liver sections, specimens were embedded in OCT containing 30% sucrose (Tissue-Tek OCT, USA) and stored at −80°C until use. Cryosections (5μm) were stained with Sudan Black to reveal lipid accumulation. Stained sections were analyzed using the NanoZoomer 2.0-RS (Hamamatsu Photonics; Japan) digital slide scanner and NDP.View software. Bright field images were captured using the Leica microscope (EC3 Camera 0.70) with Leica Acquire 1.0 software.

### Western blot analysis

Proteins were extracted from tissues by homogenizing in RIPA buffer containing protease inhibitors. Antibodies recognizing total AKT(4691S; 1:1000; 10% acrylamide gels), phospho-AKT (Ser473; 4060S; 1:1000, 10% acrylamide gels), and cytochrome oxidase (COX)-IV (4844S; 1:1000, 15% acrylamide gels) were purchased from Cell Signaling, and actin (A-4700; 1/1000) from Sigma-Aldrich. Secondary antibodies were purchased from Jackson ImmunoResearch Laboratories. Following detection of phospho-AKT, the membranes were stripped and re-probed with antibodies to total AKT or actin.

### RNA extraction and quantitative-reverse transcription-polymerase chain reaction (qRT-PCR)

Total RNA was extracted using Trizol reagent (Invitrogen), following the manufacturer’s instructions. cDNA was synthesized from 1μg of RNA using QuantiTect Reverse Transcription Kit (Qiagen; Valencia, CA, USA). Quantitative RT-PCR amplification reactions were carried out in Rotor-Gene 3000 (Corbett Life Science, Australia), MyiQ or iCycler iQ™ (Bio-Rad, CA) PCR detection systems using iQ™ SYBR® Green Supermix kit (Bio-Rad). All reactions were run in duplicates along with no template controls for each primer sets (S1 Table). Changes in mRNA expression were calculated using the difference in CT values when compared to housekeeping genes (*rpL19*, *36B4*, and *Gapdh*) and expressed relative to controls.

### Statistical Analysis

Statistical analyses were performed using GraphPad Prism 6.0 (GraphPad Software, Inc; San Diego, CA, USA) or IBM SPSS Statistics 20. Data are presented as mean ± standard error of the mean (SEM). Statistical significance (considered to be relevant when *p*<0.05) between two groups was determined by unpaired *t* test or Mann-Whitney test, as appropriate. Comparison among groups was performed using either repeated measures ANCOVA adjusted for body weight with Sidak post-hoc tests, repeated or independent non-parametric one-way ANOVA or two-way ANOVA with Dunn’s or Tukey’s post-hoc tests.

## Results

### Ablation of *Il15* protects against diet-induced obesity and insulin resistance

**Fig 1:**
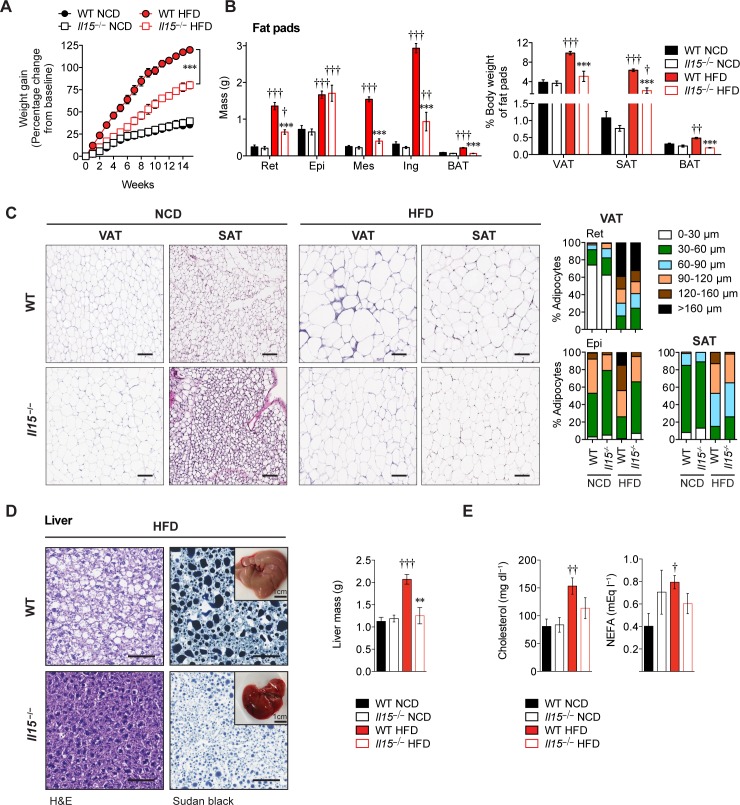
Ablation of *Il15* protects from diet-induced weight gain. **(A)** Gain in body weight of WT and *Il15*^*−/−*^ male mice fed either NCD or HFD for 16 weeks from 4 weeks of age (mean ± SEM; n = 5–11 from 2–3 independent experiments; ***p<0.001). **(B)** Mass of retroperitoneal (Ret), epididymal (Epi), mesenteric (Mes), inguinal (Ing), and interscapular BAT (mean ±SEM; n = 5–11; ***p<0.001 vs WT, ††*p*<0.01, †††*p*≦0.0001 vs NCD; left). Mass of pooled VAT (Ret, Epi and Mes), SAT (Ing) and BAT is shown as percentage of body weight (mean±SEM; n = 5–8; ***p≦0.0001 vs WT, †*p*<0.05, ††*p*<0.01, †††*p*<0.001 vs NCD; right). **(C)** H&E stained-sections of VAT (Ret) and SAT (Ing). Scale bar = 100μm. Magnification ×10. Adipocyte size distribution (cross-sectional diameter in μm; between 800–1500 adipocytes per group). **(D)** H&E and Sudan Black (for triglycerides and lipids) stained-sections of liver. Scale bar = 100μm. Magnification ×20. Representative macroscopic pictures of the livers are shown in the inset. Liver mass (*right*) of WT and *Il15*^*−/−*^ male mice maintained on NCD HFD (mean±SEM; n = 5–8; **p<0.01, vs WT, †††*p*<0.001 vs NCD. **(E)** NEFA and cholesterol levels were measured in the sera of WT and *Il15*^*−/−*^ male mice maintained on NCD HFD (mean ± SEM; n = 3–7; †*p*<0.05, ††*p*<0.01 vs NCD).

When maintained on high-fat diet (HFD), IL-15-deficient (*Il15*^***−/−***^HFD) mice showed significantly lower weight gain than control C57BL/6 (WT-HFD) mice (Fig 1A). The difference in body weight gain was also observed in *Il15*^***−/−***^ female mice and in *Apoe*^***−/−***^*Il15*^***−/−***^ mice maintained on HFD (S1 Fig). The reduced weight gain of *Il15*^***−/−***^HFD mice was not associated with any differences in growth, food consumption or significant difference in physical activity (S2A–S2C Fig). The visceral (VAT) and subcutaneous (SAT) white adipose tissue mass were significantly lower in *Il15*^***−/−***^HFD mice than WT-HFD mice with the exception of the epididymal fat pad (Fig 1B). The VAT and SAT of WT-HFD mice showed a high frequency of large adipocytes, whereas these adipose tissue depots in *Il15*^***−/−***^HFD mice predominantly contained small and mid-sized adipocytes (Fig 1C). Hepatomegaly and steatosis, which are characteristic of liver from WT-HFD mice [26], were not observed in *Il15*^***−/−***^HFD mice and their livers showed normal weight and smaller lipid-containing vacuoles with reduced staining for triglycerides (Fig 1D). The serum levels of cholesterol and non-esterified fatty acids (NEFA) were increased in WT but not in *Il15*^***−/−***^ mice that were maintained on HFD (Fig 1E). These data indicate that IL-15 plays an important role in HFD-induced fat accumulation.

**Fig 2:**
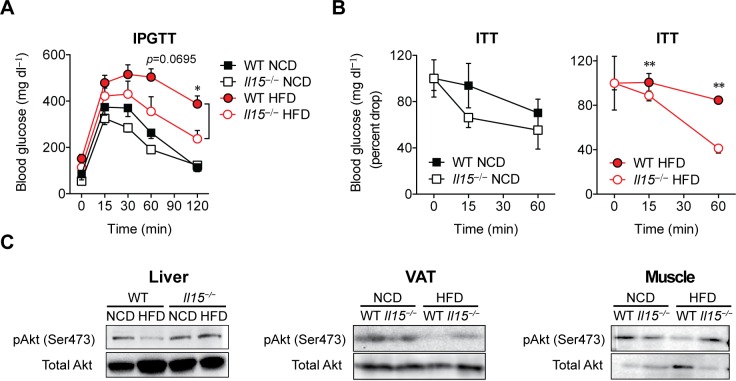
*Il15*^*−/−*^ mice maintained on HFD do not become insulin resistant. **(A)** Glucose tolerance test (GTT) following intraperitoneal glucose injection (2g/kg BW) in WT and *Il15*^*−/−*^ male mice fed either NCD or HFD (mean ± SEM; n: WT-NCD = 11; *Il15*^*−/−*^NCD = 14; WT-HFD: n = 10; *Il15*^*−/−*^HFD: n = 8; ***p<0.05 vs WT). **(B)** Insulin tolerance test (ITT) following intravenous insulin injection (1U/kg BW). ITT data is presented as percent drop from basal glucose levels. (mean±SEM; n = 3–4; *p<0.05, **p<0.01 vs WT; ††*p*<0.01 vs NCD). **(C)** Immunoblot analysis of phosphorylation of AKT on Ser473 and total AKT levels in lysates from the liver, VAT and muscle tissues.

Next we investigated the effect of *Il15* deletion on glucose utilization and insulin sensitivity. Following intraperitoneal administration of glucose, WT-HFD mice showed elevated blood glucose levels for a prolonged period, reflecting reduced glucose tolerance compared to control WT-NCD mice (Fig 2A). On the other hand, *Il15*^***−/−***^HFD mice displayed rapid clearance of blood glucose. Similarly, administration of insulin caused significantly accelerated drop in blood glucose levels in *Il15*^***−/−***^HFD mice compared to WT-HFD mice (Fig 2B). Next we examined Ser473 phosphorylation of AKT, which occurs downstream insulin receptor signaling [2]. The liver, VAT and muscle tissues of WT-HFD mice showed markedly reduced AKT phosphorylation, whereas it remained intact in *Il15*^***−/−***^HFD mice (Fig 2C). These findings indicate that lack of IL-15 is sufficient to prevent insulin resistance induced by HFD.

### Oxygen consumption is decreased in control, but not in *Il15* deficient mice maintained on HFD

**Fig 3:**
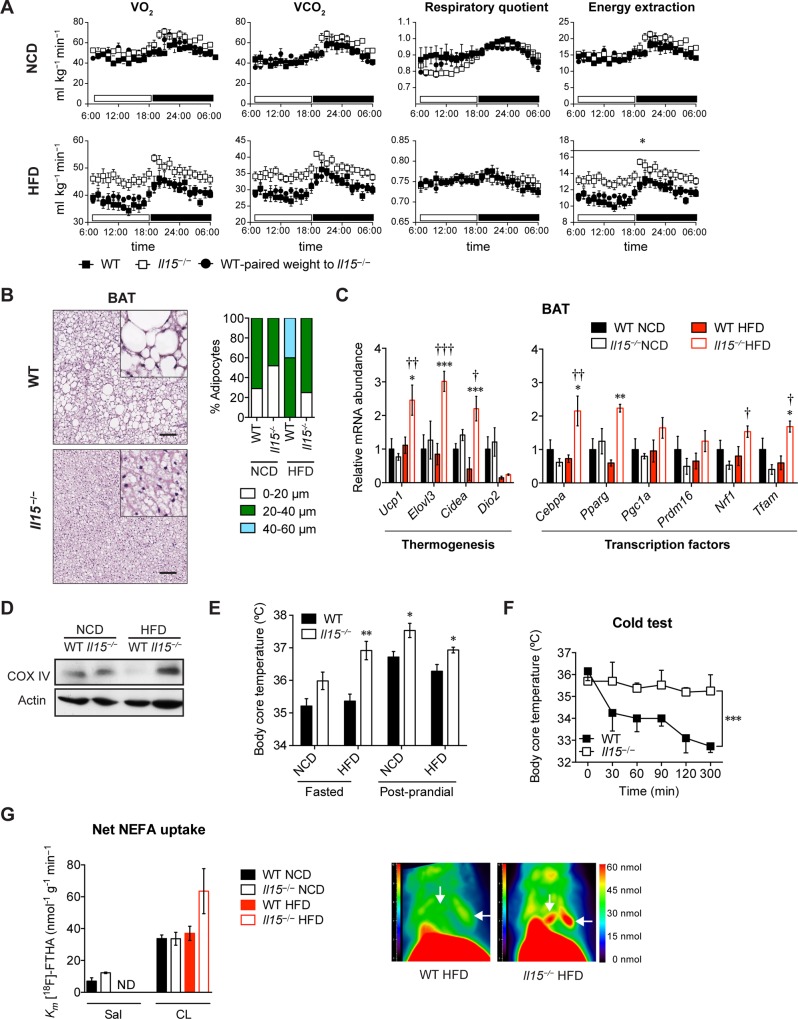
*Il15* deficiency promotes adaptive thermogenesis in the brown adipose tissue. **(A)** WT and *Il15*^*−/−*^ maintained on NCD or HFD for 16 or 7 weeks were acclimatized in metabolic chambers for 3 days followed by the measurement of oxygen consumption (VO_2_) and carbon dioxide release (VCO_2_). Energy extraction and respiratory quotient were calculated from data obtained over 3 days from 4 individual mice per group. WT mice that were on NCD or HFD for 7 weeks of age were used as weight-paired groups (weight matched to *Il15*^*−/−*^ mice on diet for 16 weeks). *p<0.05 repeated measures ANCOVA test for difference between groups adjusted for body weight. **(B)** H&E stained-sections of BAT. Scale bar = 100μm. Magnification ×10 (square is ×80). Adipocyte size distribution (*right*). **(C)** Quantitative PCR analysis of genes involved in thermogenesis in BAT of WT and *Il15*^*−/−*^ mice maintained on NCD or HFD. (mean ± SEM; n = 3–4; *p<0.05, **p<0.01, ***p<0.001 vs WT; †*p*<0.05, ††*p*<0.01, †††*p*<0.001 vs NCD). **(D)** Immunoblot analysis for cytochrome oxidase (COX) IV and actin. Data from one group of mice from 3 groups is shown. **(E)** Rectal temperature of WT and *Il15*^*−/−*^ mice (mean ± SEM; n = 3–8 mice; *p<0.05, **p<0.01 vs WT). **(F)** Rectal temperature of WT and *Il15*^*−/−*^ mice measured during a cold test (10°C, 20 h) (mean±SEM; n = 4; ***p = 0.0003). **(G)** ^18^F-fluoro-thiaheptadecanoic acid (^18^FTHA) uptake of BAT determined by positron-emission tomography-computed tomography (PET-CT) in 2–5 mice in WT and *Il15*^*−/−*^ male mice fed either NCD or HFD that were injected with CL or saline. Mice treated with saline served as controls and showed no difference in the uptake of the tracer. ND = not done. The pseudocolor bar represents quantity of the tracer (in nmol) that is detected in the BAT.

As the food consumption and locomotor activities were comparable between WT and *Il15*^***−/−***^ maintained on NCD or HFD (S2B Fig), we assessed oxygen consumption by indirect calorimetry in mice maintained on HFD or NCD for 16 weeks. As the body weight of *Il15*^***−/−***^ maintained on HFD was significantly lower than that of controls (Fig 1A), the data were corrected to body weight. We did not observe any significant differences in oxygen consumption rate, respiratory quotient or energy extraction between WT and *Il15*^***−/−***^ maintained on NCD (Fig 3A, top panel). As expected the respiratory quotient decreased in WT and *Il15*^***−/−***^ mice when maintained on HFD (Fig 3A). ToA)gined on HFDrespiratory quotient fferences *Il15-/-* HFD are not secondary to their lower body weight, we analysed WTlysed WTt secondary to their l, as their body weight was comparable to that of *Il15*^*−/−*^ maintained on HFD for 16y weight was comparable to tha*Il15*^*−/−*^ HFD remained higher compared to paired-weight WT-HFD mice. Thus,T*Il15*^*−/−*^ maintained on HFD,r compared to paired-weight WT-HFD mice. Thus,Tion rate, respiratory quotient oroxidation and utilization of lipids *in vivo*.

### *Il15* deficiency promotes adaptive thermogenesis in classical brown adipose tissue

The reduced adiposity and increased oxygen consumption of *Il15*^***−/−***^HFD mice corroborates with an increased energy expenditure in *Il15*^***−/−***^ mice compared to controls. Lipid utilization via adaptive thermogenesis in BAT represents an important process that increases energy expenditure by converting the energy from lipids into heat [6]. UCP1 present in the inner mitochondrial membrane of brown adipocytes dissipates the energy generated during oxidative phosphorylation (OXPHOS) as heat instead of generating ATP [27]. The BAT of WT-HFD mice showed increased mass and larger adipocytes akin to their WAT, whereas BAT of *Il15*^***−/−***^HFD mice retained its classical multilocular feature (Fig 3B). Accordingly, the BAT of *Il15*^***−/−***^HFD mice showed increased expression of genes associated with thermogenesis (*Ucp1*, *Evolv3* and *Cidea*), the transcription factor necessary for *Ucp1* expression (*Pparg*) and mitochondrial biogenesis (*Tfam*) (Fig 3C). Moreover, the expression of COX-IV protein, a component of the mitochondrial respiratory chain [6], was profoundly decreased in the BAT from WT-HFD but not *Il15*^***−/−***^HFD mice (Fig 3D). These data indicated that IL-15 deficiency sustained and even increased the capacity of BAT to utilize lipids during diet-induced obesity.

Next we evaluated the core body temperature, which is influenced by the thermogenic activity of BAT [6]. *Il15*^***−/−***^ mice displayed elevated core body temperature at steady state when compared to control mice (Fig 3E). Moreover, *Il15*^***−/−***^ mice maintained their core body temperature for longer periods than control mice following prolonged exposure to cold (20h at 10°C) (Fig 3F). As the thermogenic activity of BAT results mainly from fatty acid utilization [28] and *Il15*^***−/−***^HFD mice do not extensively accumulate lipids in BAT (Fig 3B), we measured fatty acid uptake by PET/CT using the positron-emitting fatty acid analog ^18^FTHA. An acute 30-min exposure to the β3 agonist CL-316243 (CL) showed a tendency for increased NEFA uptake by the BAT in *Il15*^***−/−***^HFD mice compared to WT-HFD mice (Fig 3G). Thus, the increased thermogenic activity of BAT in *Il15*^***−/−***^ mice is likely to result from the utilization of circulating lipids.

### *Il15* deficiency promotes the browning program in WAT

**Fig 4:**
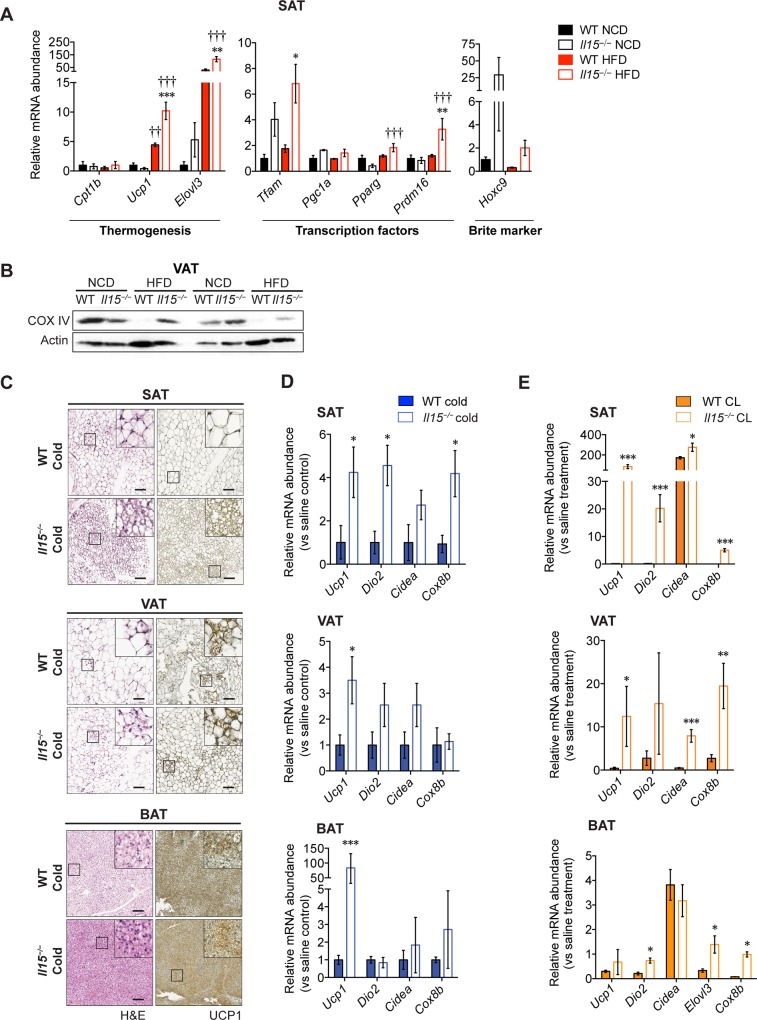
*Il15* deficiency promotes the browning program of white adipose tissues. **(A)** Quantitative PCR analysis of genes involved in thermogenesis in the SAT of WT and *Il15*^*−/−*^ male mice fed either NCD or HFD (mean ± SEM; n = 3–4; **p<0.01, ***p<0.001 vs WT; †††*p*<0.001 vs NCD). **(B)** Immunoblot analysis for cytochrome oxidase (COX) IV and actin in VAT of 2 representative of 3 groups are shown. **(C)** H&E and UCP1 stained-sections of SAT, VAT and BAT of mice exposed to a cold test. Scale bar = 100 μm. Magnification ×10 (inset ×80). **(D)** Quantitative PCR analysis of genes involved in thermogenesis in the SAT, VAT and BAT of cold-exposed WT and *Il15*^*−/−*^ male mice (mean ± SEM; n = 3–4; *p< 0.05). **(E)** Quantitative PCR analysis of genes involved in thermogenesis in the SAT, VAT and BAT of CL316243-treated WT and *Il15*^*−/−*^ male mice (mean ± SEM; n = 3–4; *p<0.05, **p<0.01, ***p<0.001 vs WT).

Brite or beige adipocytes, displaying metabolic and molecular signatures similar to brown adipocytes can arise in WAT [29–31]. Consistent with a potential role of IL-15 in modulating the browning of WAT, SAT from *Il15*^***−/−***^HFD mice showed increased expression of genes associated with thermogenesis (*Ucp1*, *Elovl3*) or differentiation toward a beige phenotype (*Prdm16*) compared to control mice [25] (Fig 4A). Similarly to BAT, VAT from WT-HFD mice showed profound decrease in COX-IV levels, whereas *Il15*^***−/−***^ mice appear to be protected from the HFD-induced decrease in COX-IV expression (Fig 4B). The protein and mRNA levels of *Ucp1* and the expression of other genes implicated in thermogenesis (*Dio2*, *Cidea* and *Cox8b*) were significantly elevated in the SAT of cold-exposed or CL-treated *Il15*^***−/−***^ mice (Fig 4C–4E). On the other hand, increased *Ucp1* induction in the BAT of *Il15*^***−/−***^ mice was observed following cold exposure but not after CL treatment (Fig 4D and 4E, bottom panels). As *Ucp1* upregulation following CL treatment occurs within 6–24h, its lack of induction following 7 days of CL administration may result from acclimatization [23, 32, 33]. Nevertheless, increased induction of thermogenic genes in the SAT of *Il15*^***−/−***^ mice following cold exposure or prolonged CL treatment indicates that IL-15 not only inhibits the thermogenic functions of BAT but can also compromise the activity of brite cells in WAT.

### IL-15 directly compromises the thermogenic program in brown and white adipocytes differentiated in vitro

**Fig 5:**
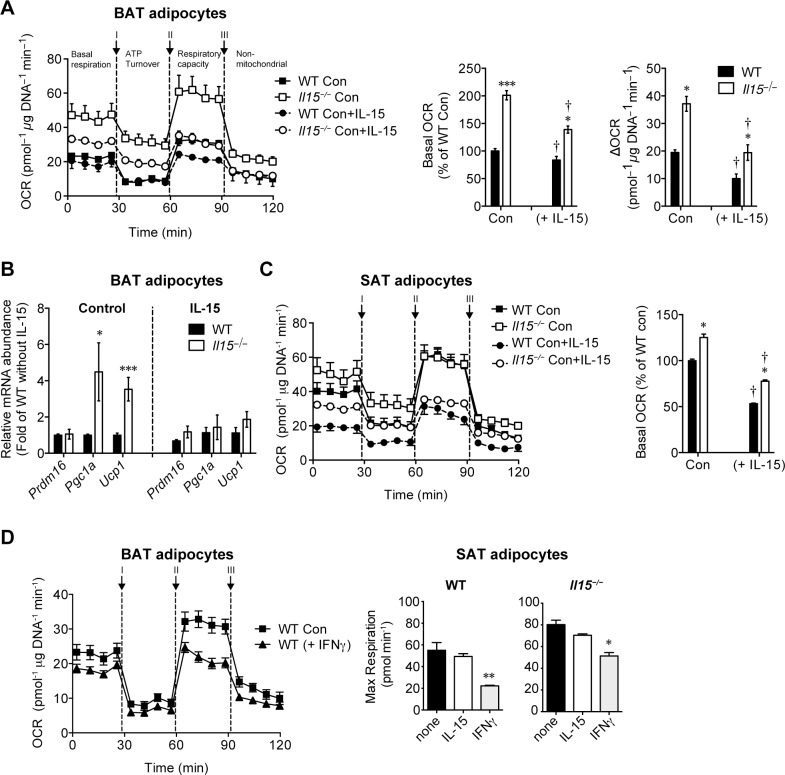
IL-15 directly compromises the thermogenic program of brown and white adipocytes *in vitro*. **(A)** Measurement of oxygen consumption rates (OCR) of primary in vitro differentiated adipocyte cultures established from BAT stromal fraction of WT and *Il15*^*−/−*^ mice differentiated with or without IL-15 (mean±SEM; n = 10–12 performed in 2 independent experiments; *p<0.05, ***p<0.0001 vs WT; †*p*<0.05 vs Control). The bars in the right panels indicate the basal and delta OCR. **(B)** Quantitative PCR analysis of thermogenic genes of primary BAT adipocytes from WT or *Il15*^*−/−*^ (mice (mean ± SEM; WT: n = 6–16 performed in 3–8 independent experiments, *Il15*^*−/−*^: n = 4–14 performed in 3–8 independent culture experiments; *p<0.05, ***P = 0.0002). **(C)** Measurement of OCR of primary adipocytes in the presence of IL-15 obtained from SAT stromal fraction of WT and *Il15*^*−/−*^ mice differentiated with or without IL-15 (mean±SEM; n = 10–12 performed in 2 independent experiments; *p<0.05 vs WT; †*p*<0.05 vs Control). The bars in the right panel represent the basal OCR. **(D)** Measurement of OCR of primary BAT and SAT adipocytes of WT mice in the presence or absence of IFNγ (10ng/ml) for 18 hours prior to the assay (mean±SEM; n = 10–12 performed in 2 independent experiments; *p<0.05; **p<0.01 vs Control).

The results described above do not distinguish the direct effects of IL-15 on adipocytes from the indirect effects mediated by the induction of other inflammatory mediators *in vivo*. To determine whether IL-15 directly alters mitochondrial functions in adipocytes, we evaluated the O_2_ consumption rates (OCR) as a measure of oxidative phosphorylation (OXPHOS) in brown adipocytes differentiated *ex vivo* from WT and *Il15*^***−/−***^ mice in the presence or absence of exogenously added IL-15. The basal OCR and the spare respiratory capacity (ΔOCR; the difference between basal and maximal respiratory rates) were higher in in vitro differentiated brown adipocytes from *Il15*^***−/−***^ mice compared to controls (Fig 5A). Addition of IL-15 during the *in vitro* differentiation process suppressed the basal OCR in brown adipocytes from *Il15*^***−/−***^ and WT mice equally, suggesting that the lower basal OCR in WT adipocytes compared to *Il15*^***−/−***^ cells could be attributed to endogenous IL-15 production (Next section). The increase in oxygen consumption is not restricted to adipocytes, as similar differences were observed in primary hepatocytes isolated from *Il15*^***−/−***^ mice (S3 Fig). IL-15 supplementation throughout the differentiation period also suppressed the OCR in brite adipocytes from the SAT of *Il15*^***−/−***^ and WT mice (Fig 5C). In contrast to the effects of long-term IL-15 treatment, short-term exposure (18h) to IL-15 did not suppress the maximal respiration in white adipocytes from *Il15*^***−/−***^ or WT mice, while addition of IFNγ caused a marked reduction (Fig 5D). These data suggest that long-term exposure to IL-15 might alter the OXPHOS in adipocytes.

**Fig 6:**
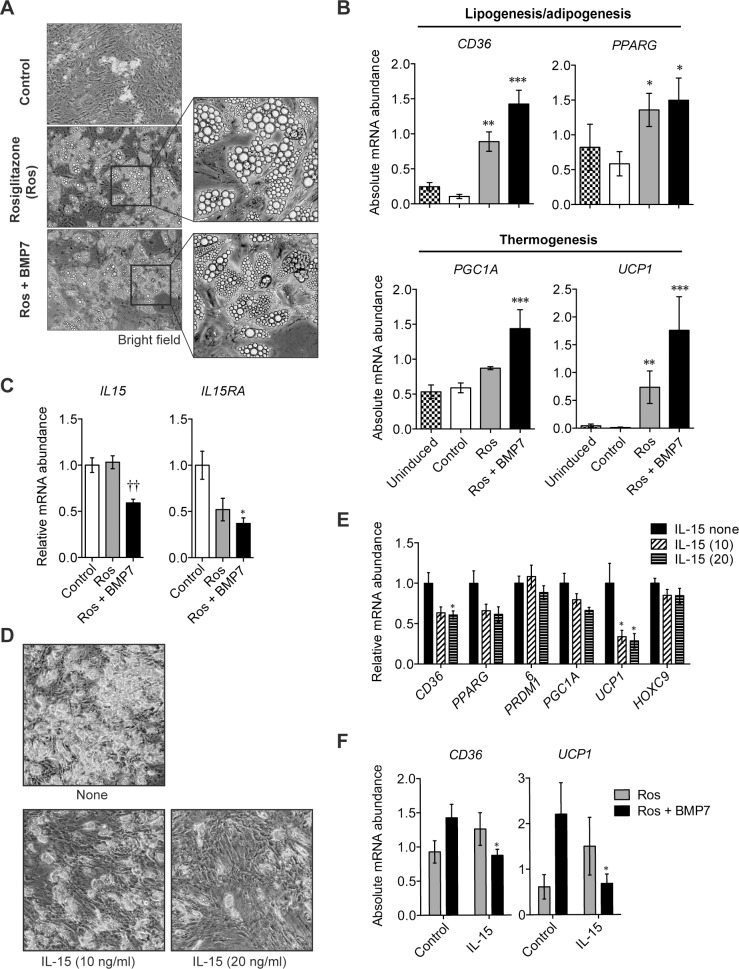
*In vitro* characterization of human beige adipocytes. **(A)** Representative bright field microscopy pictures of human primary pre-adipocytes differentiated for 21 days using the standard cocktail of adipocyte differentiation (Control) in the presence of rosiglitazone (Ros, 100nM) ± BMP7 (2nM). Magnification ×10. **(B)** Quantitative PCR analysis of gene expression of markers of lipogenesis/adipogenesis and thermogenesis of human SAT adipocytes (mean±SEM; n = 5–8 performed in 3 independent culture experiments, 3 donors; *p<0.05, **p<0.01, ***p<0.001 vs Control). **(C)** Quantitative PCR analysis of *Il15* and *IL15RA* expression of human SAT primary pre-adipocytes differentiated for 14 days with rosiglitazone (Ros, 100nM) + BMP7 (2nM) in the presence or absence of IL-15 (mean±SEM; n = 8–9 in 3 independent experiments, 3 donors; *p<0.05 vs WT; ††*p*<0.01 vs Rosiglitazone). **(D)** Bright field microscopy pictures of primary adipocytes from SAT of one representative donor differentiated as described in (**C**) ± IL-15 (10 or 20ng/ml). (**E**) Quantitative PCR analysis of brown or beige gene marker expression (mean±SEM; n = 8–9 in 3 independent experiments, 3 donors; *p<0.05 vs unstimulated adipocytes). **(F)** IL-15 suppressed the expression of *UCP1* (*p* = 0.045 vs Control) and *CD36* expression (*p* = 0.036 vs Control) in adipocytes differentiated in the presence of BMP7.

To test whether IL-15 influences the thermogenic program in human adipocyte precursors, we generated brite cells from the stromal cells of human SAT using the differentiation factors (BMP7 and Rosiglitazone) [34] (Fig 6A and 6B). Whereas the expression of endogenous *IL15* progressively decreased during the differentiation of human brite adipocytes (Fig 6C), addition of exogenous IL-15 hindered the accumulation of lipid droplets (Fig 6D). The presence of IL-15 during the differentiation of human brite cells also reduced the expression of genes involved in thermogenesis (*CD36*, *UCP1*) (Fig 6E and 6F). Although IL-15 did not influence the expression of *PRDM16* or *HOXC9* (Fig 6E), we do not exclude the possibility that IL-15 may influence the differentiation/development of brown/beige adipocytes *in vivo*. Nevertheless, the elevated expression of thermogenic genes in the adipose tissues of IL-15-deficient mice maintained on HFD (Fig 3B) or upon treatment with CL or exposure to cold (Fig 4C–4E), and the inhibition of these genes by IL-15 in human beige adipocytes (Fig 6E and 6F) strongly support the idea that IL-15 inhibits the thermogenic program in brown/beige adipocytes.

### IL-15 is required for obesity-induced inflammation

**Fig 7:**
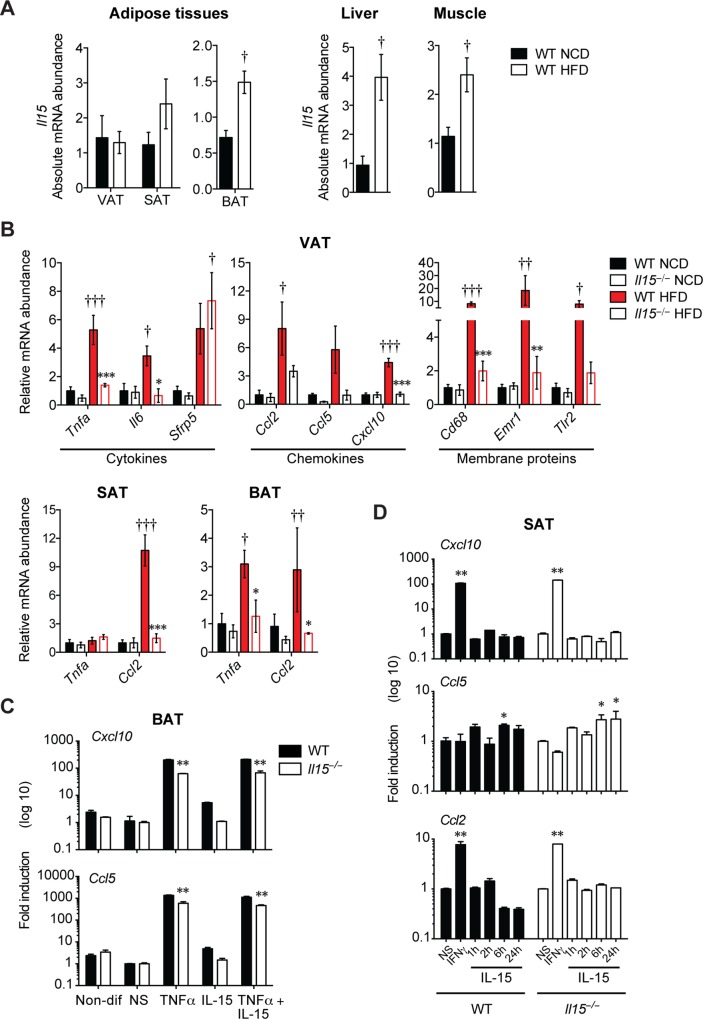
IL-15 is required for obesity-induced inflammation. **(A)** Quantitative PCR analysis of *Il15* expression of adipose tissues, liver and muscle of NCD or HFD -fed WT mice (mean±SEM; n = 3–4; †*p*<0.05 vs NCD). **(B)** Quantitative PCR analysis of inflammatory genes of VAT, SAT and BAT of WT and *Il15*^*−/−*^ male mice fed either NCD or HFD (mean±SEM; WT: n = 3–4; *p<0.05, **p<0.01, ***p<0.001 vs WT; †*p*<0.05, ††*p*<0.01, †††*p*<0.001 vs NCD). **(C)** Quantitative PCR analysis of chemokine genes in murine brown adipocytes differentiated from SVC of BAT from WT or *Il15*^*−/−*^ mice untreated, or treated with TNFα (20ng/ml), IL-15 (20ng/ml) or TNFα+IL-15 during the last 18 hours of culture (mean±SEM; n = 3 independent experiments; **p<0.01, vs non-stimulated controls of the same genotype). **(D)** Quantitative PCR analysis of genes associated with thermogenesis in murine beige adipocytes differentiated from SVC of SAT from WT and *Il15*^*−/−*^ mice untreated, or treated with TNFα (20ng/ml), IL-15 (20ng/ml) or TNFα+IL-15 during the differentiation process (mean±SEM; n = 3 independent experiments; **p<0.01, vs non-stimulated controls of the same genotype).

IL-15 is an inflammatory cytokine that modulates the functions of both innate and adaptive immune cells [17]. WT mice maintained on HFD showed significantly increased expression of *Il15* in the BAT, liver and skeletal muscles and to a lesser extent in the SAT, but not in the VAT, compared to WT-NCD mice (Fig 7A). To determine whether IL-15 promotes inflammation besides directly modulating thermogenic functions, we evaluated cytokine and chemokine gene expression in adipose tissues. Even though HFD did not increase *Il15* expression in the VAT of WT-HFD mice (Fig 7A), the VAT of *Il15*^***−/−***^HFD mice showed significantly reduced expression of inflammatory cytokines (*Tnfa*, *Il6*) and chemokine (*Cxcl10*) and a marker associated with macrophage (*Cd68*) (Fig 7B). These data suggest that the VAT is a major source of IL-15-dependent inflammatory mediators that are induced in response to dietary fat overload. Next, we examined whether IL-15 directly modulated the induction of chemokine genes in brown and beige adipocytes. While TNFα strongly induced the expression of *Cxcl10* and *Ccl5* in in vitro differentiated brown adipocytes, IL-15 failed to induce these genes in BAT but elicited a modest induction of *Ccl5* in beige adipocytes from SAT (Fig 7C and 7D). Collectively, these data suggest that IL-15 induced by HFD in adipose tissues increase the expression of inflammatory mediators, which in turn perpetuate the inflammatory cascade and promote the development of metabolic syndrome.

## Discussion

Inflammatory cytokines produced during lipid overload promotes insulin resistance by inhibiting the pathways downstream of insulin receptor signaling and by perpetuating the inflammatory cascade [1, 3]. Here we show that the pro-inflammatory cytokine IL-15 also inhibits adaptive thermogenesis in adipose tissues. *Il15*-deficient mice fed with HFD exhibit an increased thermogenic capacity in brown and beige fat cells that correlates with resistance to diet-induced weight gain and loss of insulin sensitivity. Additionally, these mice were protected from inflammation and other manifestations of metabolic syndrome such as hepatic steatosis [35] and dyslipidemia. Our observations complement another recent study that showed that IL-15Rα deficient mice are protected from obesity [36].

In contrast to our findings, several studies have reported a beneficial role of IL-15 in obesity. Treatment of genetically obese mice (*ob/ob*) with IL-15 has been shown to reduce body fat [37]. Subsequent observation of IL-15 expression by skeletal muscles following exercise led to the suggestion that the beneficial effect of exercise could be partly attributed to IL-15 [20]. However, circulating IL-15 levels peak 10min after exercise before returning to basal level within 1 hour [38]. Moreover, systemic increase in IL-15 resulted in enhanced apoptosis of skeletal muscle cells [39]. Therefore, it is likely that the beneficial effect of exercise may be mediated by other factors like Irisin or Meteorin-like hormone that are also produced by muscles and help to reduce insulin resistance [40, 41], rather than by IL-15.

In contrast to our findings, IL-15-deficient mice were reported to gain weight upon aging that was reversed by IL-15 treatment [19, 42–44]. The same study also showed that IL-15 transgenic mice show resistance to weight gain when maintained on regular diet, upon ageing. We do not have a plausible explanation for these contrasting observations on *Il15*^***−/−***^ mice maintained on HFD, which could arise from the influence of several factors including microbiota [45]. Similarly, transgenic expression of IL-15 in the muscles and in circulation increased the oxidative energy metabolism in muscles, without any obvious increase in circulating inflammatory cytokines [46]. In contrast, whole body IL-15 transgenic mice show increased inflammation and develop leukemia [47]. It is possible to reconcile these observation as follows: Overexpression of IL-15 in immune cells and other cell types associated with the immune system such as intestinal epithelial cells can overtly activate NK cells and cause inflammation [48]. Also, IL-15 in circulation is essentially complexed with IL-15Rα that is shed from the cell surface and is not biologically active as soluble IL-15:IL-15RαFc is active only in mice with intact Fc receptor [49, 50]. As the cleaved soluble IL-15:IL-15Rα complex cannot become surface bound, it may not be able to trans-present IL-15 to cells expressing the βγ complex and hence may not be biologically active. Thus over-expression of IL-15 in the muscle may not lead to increase in the inflammatory phenotype. Nevertheless, other lines of evidence support our contention that IL-15 plays a pathogenic role in obesity. First, IL-15Rα-deficient mice lacking the ligand-binding subunit of the IL-15 receptor complex display increased locomotor activity, lean body mass and increased fat utilization [36, 51], indicating that IL-15 signaling attenuates energy expenditure, similarly to our findings on IL-15-deficient mice.

**Fig 8:**
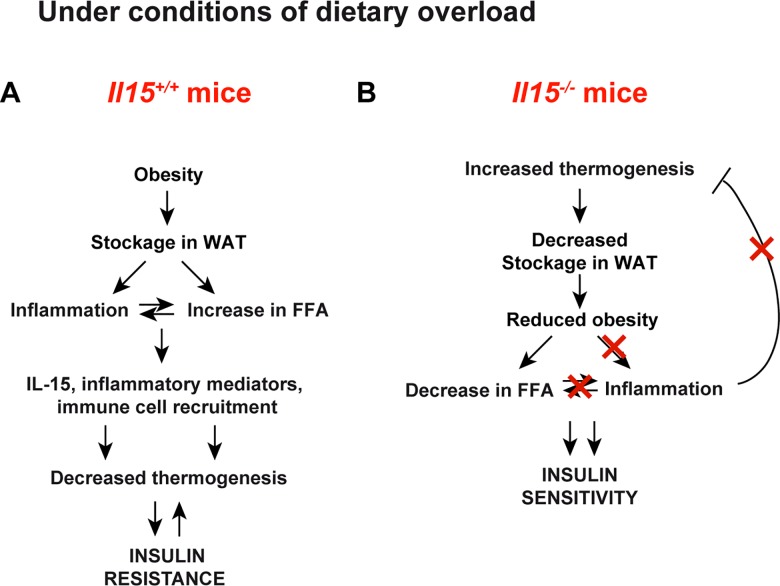
**Proposed mechanism of action of IL-15 in obesity (A**) Under conditions of dietary lipid overload, excess calories are stored in the white adipose tissues (WAT) resulting in obesity. This results in an increase in circulating free fatty acids (FFA) and increased production of inflammatory mediators. Increased IL-15 results in the production of chemokines and other inflammatory mediators and recruitment and activation of immune cells. Many of the inflammatory mediators including IL-15 inhibit adaptive thermogenesis and promote insluin resistance. (**B**) In the absence of IL-15 adaptive thermogenesis is increased. As consequence stockage of lipids is decreased with a concomitant reduction in FFA. There is minimal induction of inflammation. Adaptive thermogenesis is not inhibited and insulin sensitivity is maintained.

Although IL-15 transgenic mice do not become obese [19], this model may not be suitable for studying the effect of IL-15 on obesity, as they develop fatal lymphocytic leukemia [47]. Similarly, studying the effect of IL-15 treatment on obesity may be confounded by the profound effects of IL-15 in activating the immune system. IL-15 induces the expression pro-inflammatory cytokines such as IL-6 and TNFα from macrophages [17] that are implicated in the pathogenesis of obesity-associated metabolic syndrome [1]. However, TNFα can also cause cachexia that may explain the decrease in the body fat of rodents treated with IL-15 [52]. IL-15 treatment also stimulates the immune system to fight against solid tumors by stimulating CD8^+^ T lymphocytes [17]. In fact, clinical trials on IL-15 therapy for cancer treatment resulted in cytokine storm [53]. Different studies have shown elevated levels of circulating IL-15, IL-6, CRP and IL-1Rα have been observed in obese individuals [21, 54, 55]. Circulating IL-15 levels are shown to be higher in overweight patients exhibiting increased abdominal adiposity and suffering from coronary artery disease [21]. Increased expression of *Il15* gene in the SAT of wild-type mice maintained on HFD (Fig 7A) supports the notion that adipose tissue depots can also be a source of IL-15 [21]. The elevated circulating levels of NEFAs in obesity may contribute to increased IL-15 gene expression in adipose tissues as free fatty acids can activate pathogen-sensing systems like Toll-like receptors (TLRs), leading to the production of pro-inflammatory cytokines [3]. Induction of chemokines that can further recruit other inflammatory cells such as macrophages and CD8^+^ T cells to the obese adipose tissue could perpetuate chronic inflammation (Fig 8). The above arguments support the pathogenic role of IL-15 in metabolic syndrome via induction and perpetuation of the inflammatory response. On the other hand, we cannot rule out the possibility that the reduced inflammation and maintenance of the normal metabolic phenotype is secondary to the reduced weight gain.

It has also been reported that treatment of rats on normal diet with IL-15 upregulates *Ucp1* and *Ucp3* mRNA levels in BAT, accompanied with 24% and 35% decrease in BAT and WAT mass, respectively [52]. These observations stand in sharp contrast to our findings on IL-15-deficient mice maintained on HFD, as well as those of Pistilli *et al*., on IL-15Rα-deficient mice [51]. The resistance to weight gain observed in *Il15*-deficient mice, along with their increased body temperature, resistance to hypothermia and marked up-regulation of thermogenic genes in BAT, strongly indicate increased energy expenditure in these mice. However, IL-15 does not appear to interfere with the molecular pathway determining the adipocyte-browning program involving PRDM16 [25]. In addition, the diminution of *Cd36* expression by IL-15 in primary adipocytes, suggests that IL-15 may regulate free fatty acid transport across the plasma membrane. In agreement, *Il15*^***−/−***^ mice exhibit higher NEFA uptake in their BAT *in vivo* (Fig 3G). Altogether, these data indicate that the IL-15-mediated inhibition of thermogenic functions may result mainly from impaired lipid uptake, which is the limiting step in the thermogenic function of brown adipocytes [56].

Previous reports have suggested a role for IL-15 in promoting oxidative phosphorylation and *Cpt1a* expression in memory subset of CD8^+^ T cells [57]. These results are in direct contrast with the results presented here in adipocytes and hepatocytes (Fig 5 and S3 Fig). Absence of endogenous IL-15 enhanced various aspects related to oxidative phosphorylation, more particularly oxygen consumption by mitochondria in brown and white adipocytes and hepatocytes. The differences may rely on the ability of these cell types to produce IL-15. CD8^+^ T lymphocytes do not produce IL-15 but can respond efficiently to IL-15. As inflammatory phenotype is associated with a shift towards glycolysis, it will be important to understand the mechanisms by which IL-15 regulates OXPHOS in a tissue specific manner.

At present we do not know the origin of IL-15 in the tissues. The increased infiltration of immune cells, in particular macrophages, in the liver and VAT (up to 50%, [3]) can be the potential source for IL-15. The fact that only membrane bound IL-15 is active and not the IL-15 in circulation complicates the analysis of IL-15 in tissues [58]. However, in contrast to TNFα or IFNγ, the direct effects of exogenous IL-15 on the inflammatory process are at most modest (Fig 7). One of the reasons could be the general difficulty that is associated with studies involving IL-15 [58]. While exogenous IL-15 activates lymphocytes efficiently, IL-15 acts in an autocrine manner in macrophages and DCs [16]. Increase in pro-inflammatory mediators such as IFNγ can induce the expression of IL-15 in the adipose tissue to modulate the thermogenic program. Similarly, it is also possible that the protective effect conferred by the absence of endogenous IL-15 can be overcome by other pro-inflammatory mediators. We observed that TNFα or IFNγ induced the expression of chemokines in IL-15 deficient adipocytes (Fig 7). Thus, factors affecting the basal inflammatory status can contribute to the variations observed in the protection from HFD-induced weight gain in IL-15 deficient animals reported by others. It is possible that the modulation of oxidative phosphorylation and the thermogenic program may be indirect effects of IL-15 on metabolism whereby IL-15 affects the differentiation process and the pattern of gene expression. In addition, induction of IL-15 in macrophages and dendritic cells as a consequence of lipid overload can induce the production of pro-inflammatory cytokines such as TNFα or IFNγ from tissue resident immune cells. As these cytokines can upregulate the expression of chemokines in the target tissues and potentiate the recruitment of additional immune cells, the vicious loop of inflammation gets established to induce the pathology without any further requirement for IL-15 per se. Deletion of IL-15 in *ob/ob* or *db/db* for obesity and *Apoe*- and *Ldlr*- deficient mice for atherosclerosis will help to delineate the underlying mechanisms.

The role of inflammatory factors in the regulation of metabolism may have evolved to counteract the anabolic functions of insulin, in order to mobilize nutrients to fuel the immune response [1, 3]. IL-15 induced following infection can activate the inflammatory cascade by stimulating the expression of chemokines in the infected tissues while inhibiting energy wastage by thermogenesis in BAT, as shown in this study. However, the same functions are detrimental when IL-15 may be induced in response to dietary lipid overload (Fig 8). Thus, neutralization of IL-15 or blockade of IL-15 signaling may prevent inflammation and activate brown/beige function during nutrient excess. Our study suggests that IL-15 may be a therapeutic target not only in autoimmune type 1 diabetes [17, 59, 60], but also in obesity.

## Supporting Information

S1 Data Raw data for figures presented in this manuscript. (XLSX)

S1 Fig Ablation of *Il15* protects female mice and *Apoe*-deficient mice diet-induced weight gain. **(A)** Gain in body weight of female *Il15*-deficient and (B) *Apoe*-deficient- *Il15*-deficient mice fed either NCD or HFD for 16 weeks from 4 weeks of age (mean±SEM; n = 8 from 2–3 independent experiments; *p<0.05).
(TIF)

S2 Fig **Anthropometric and Physiological Parameters of *Il15***^***−/−***^ **Mice (A**) Representative photos of WT and *Il15*^*−/−*^ mice fed NCD or HFD and their body length (mean ± SEM; n = 3–6). **(B)** Total ambulatory and fine movement counts during 24 h (mean ± SEM; n = 3–4; *P < 0.05). AU, arbitrary units. **(C)** Food intake and feeding efficiency (mean ± SEM; n = 4; *P < 0.05 vs WT; †††*P* < 0.001 vs NCD).
(TIF)

S3 Fig Endogenous IL-15 alters oxygen consumption in hepatocytes *in vitro*. Measurement of oxygen consumption rates (OCR) of primary hepatocytes of WT and *Il15*^*−/−*^ mice (mean±SEM; n = 10–12 performed in 3 independent experiments; *p<0.05, vs WT). The bars in the right panel indicate the delta OCR.
(TIF)

S1 Table Primer-sequences used for real-time qPCR analysis of gene expression. (DOCX)

## Abbreviations

BAT: brown adipose tissue
GTT: glucose tolerance test
HFD: high fat diet
IGT: impaired glucose tolerance
IHC: immunohistochemistry
ITT: insulin tolerance test
NCD: normal chow diet
NEFA: non-esterified fatty acids
OCR: oxygen consumption rate
OXPHOS: oxidative phosphorylation
SAT: subcutaneous adipose tissue
SVC: stromal vascular fraction cells
T2D: Type 2 diabetes
UCP1: Uncoupling protein 1
VAT: visceral adipose tissue
WAT: white adipose tissue
WT: wild-type
