# Correction: Deficiency of Interleukin-15 Confers Resistance to Obesity by Diminishing Inflammation and Enhancing the Thermogenic Function of Adipose Tissues

**DOI:** 10.1371/journal.pone.0166537

**Published:** 2016-11-08

**Authors:** Gregory Lacraz, Volatiana Rakotoarivelo, Sebastien M. Labbé, Mathieu Vernier, Christophe Noll, Marian Mayhue, Jana Stankova, Adel Schwertani, Guillaume Grenier, André Carpentier, Denis Richard, Gerardo Ferbeyre, Julie Fradette, Marek Rola-Pleszczynski, Alfredo Menendez, Marie-France Langlois, Subburaj Ilangumaran, Sheela Ramanathan

There is an error in the last three sentences in the paragraph “Oxygen consumption is decreased in control, but not in *Il15* deficient mice maintained on HFD” of the Results section. The correct sentences are: To rule out that metabolic changes observed in Il15-/- HFD are not secondary to their lower body weight, we analysed WT mice following 7 weeks on HFD, as their body weight was comparable to that of *Il15*^*−/−*^ maintained on HFD for 16 weeks. Oxygen consumption in *Il15*^*−/−*^ HFD remained higher compared to paired-weight WT-HFD mice. Thus, *Il15*^*−/−*^ maintained on HFD, but not NCD show elevated oxygen consumption, indicative of mitochondrial beta-oxidation and utilization of lipids *in vivo*.
