# Supplementary figures and images for: Deficiency of Interleukin-15 Confers Resistance to Obesity by Diminishing Inflammation and Enhancing the Thermogenic Function of Adipose Tissues
Source: PLoS One. 2016 Sep 29;11(9):e0162995. doi: 10.1371/journal.pone.0162995 (PMC5042499; doi:10.1371/journal.pone.0162995)

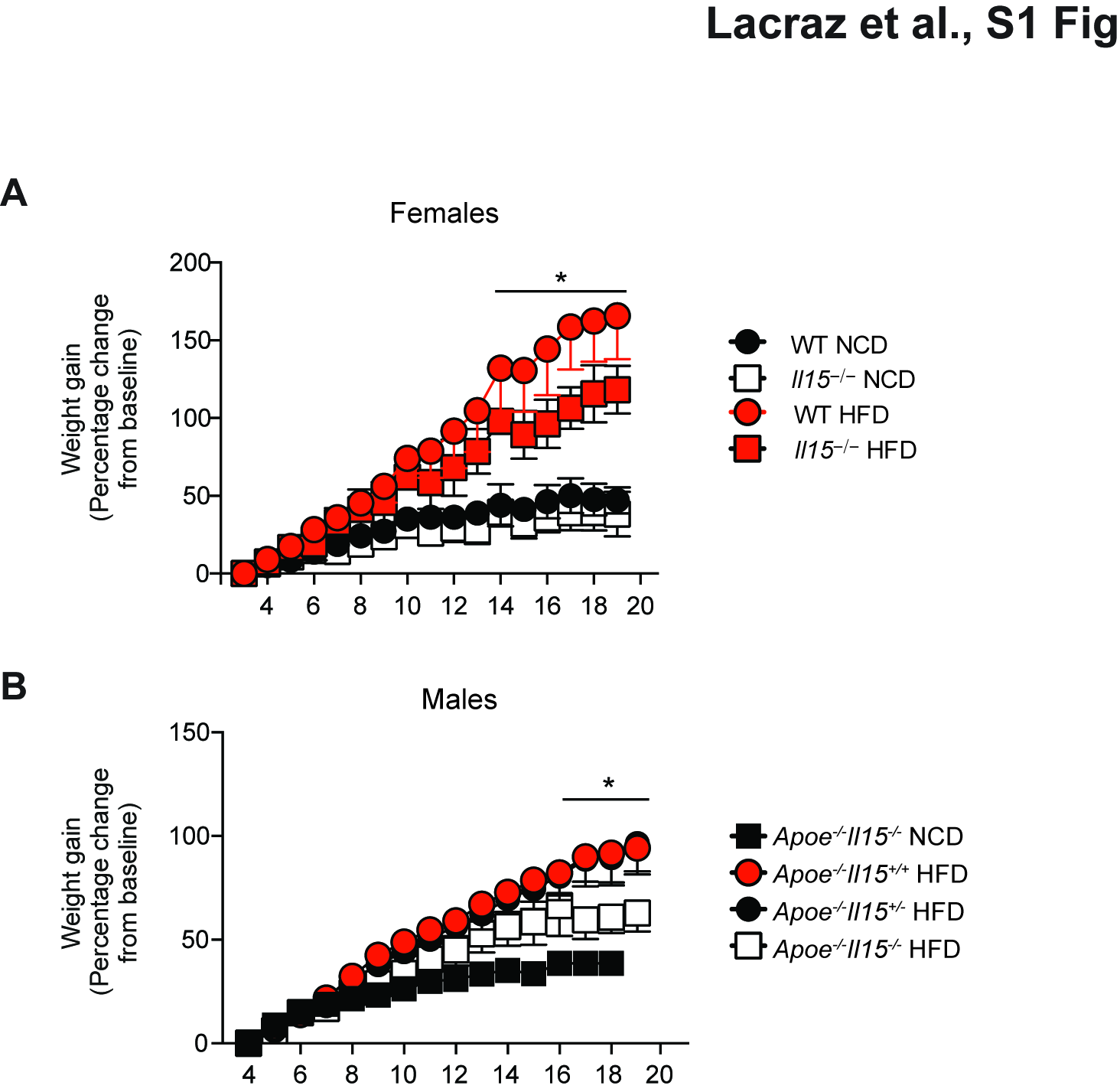

Supplement: S1 Fig — (A) Gain in body weight of female Il15-deficient and (B) Apoe-deficient- Il15-deficient mice fed either NCD or HFD for 16 weeks from 4 weeks of age (mean±SEM; n = 8 from 2–3 independent experiments; *p<0.05). (TIF) [file pone.0162995.s002.tif]

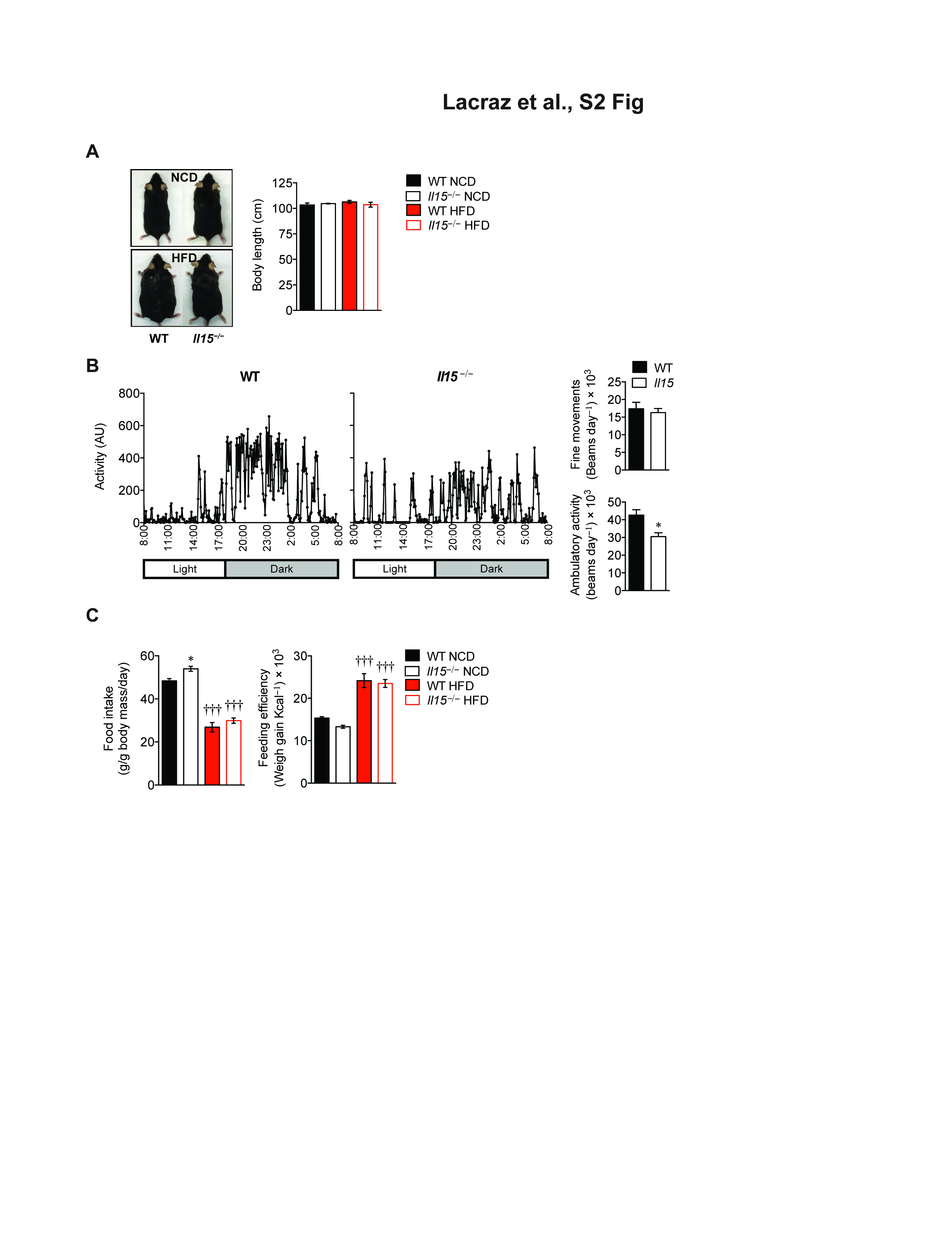

Supplement: S2 Fig — Anthropometric and Physiological Parameters of Il15−/− Mice (A) Representative photos of WT and Il15−/− mice fed NCD or HFD and their body length (mean ± SEM; n = 3–6). (B) Total ambulatory and fine movement counts during 24 h (mean ± SEM; n = 3–4; *P < 0.05). AU, arbitrary units. (C) Food intake and feeding efficiency (mean ± SEM; n = 4; *P < 0.05 vs WT; †††P < 0.001 vs NCD). (TIF) [file pone.0162995.s003.tif]

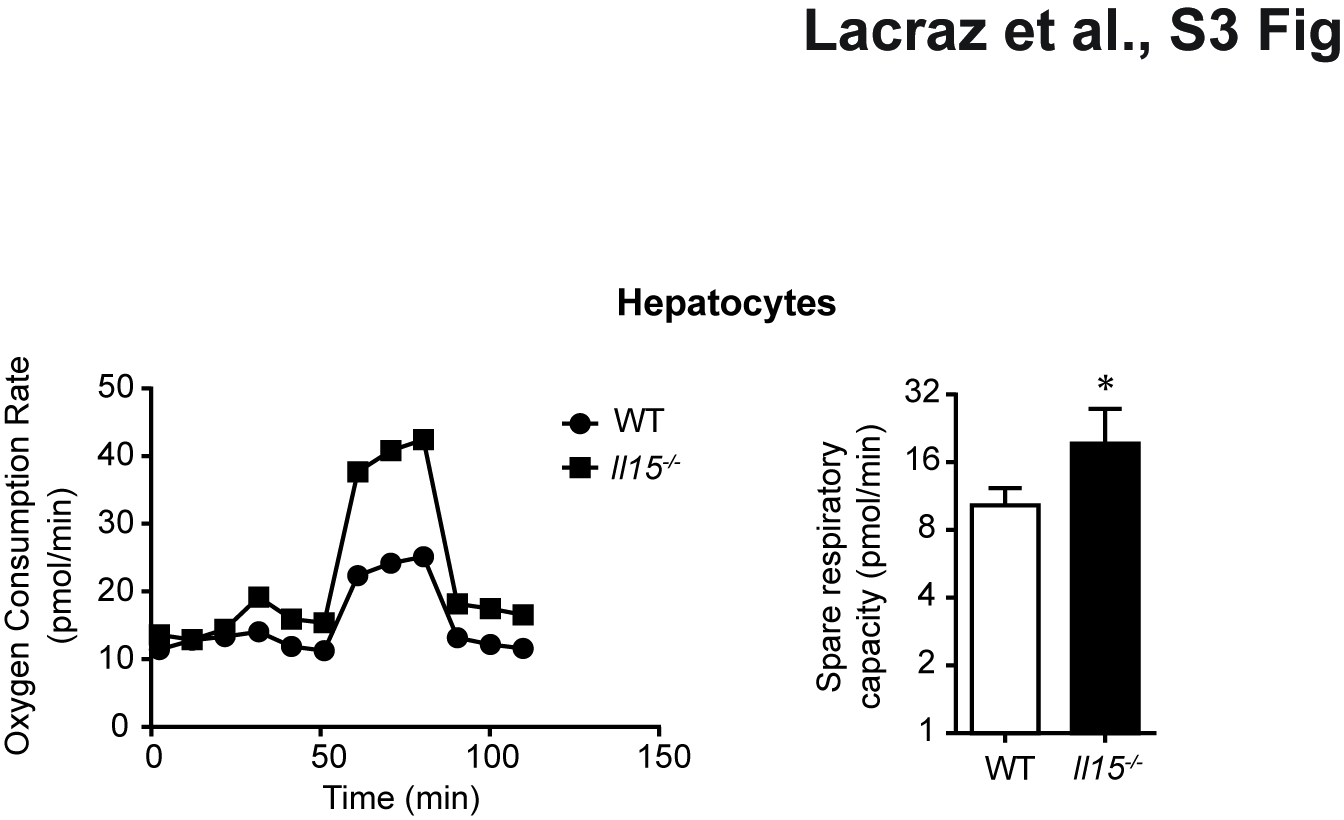

Supplement: S3 Fig — Measurement of oxygen consumption rates (OCR) of primary hepatocytes of WT and Il15−/− mice (mean±SEM; n = 10–12 performed in 3 independent experiments; *p<0.05, vs WT). The bars in the right panel indicate the delta OCR. (TIF) [file pone.0162995.s004.tif]
