# Supplementary material for: Deficiency of Interleukin-15 Confers Resistance to Obesity by Diminishing Inflammation and Enhancing the Thermogenic Function of Adipose Tissues
Source: PLoS One. 2016 Sep 29;11(9):e0162995. doi: 10.1371/journal.pone.0162995 (PMC5042499; doi:10.1371/journal.pone.0162995)
Supplement: S1 Table — (DOCX) [file pone.0162995.s005.docx]

**S1 Table: Primer-sequences used for real-time qPCR analysis of gene expression.**

| **Gene** | **Species** | **Accession number** |  | **Sequence** |
| --- | --- | --- | --- | --- |
| ***Acadl*** | Mus Musculus | NM_007381.3 | Forward  Reverse | tggggacttgctctcaaca  ggcctgtgcaattggagta |
| ***Acadm*** | Mus Musculus | NM_007382.4 | Forward  Reverse | tgtcgaacacaacactcgaaa  ctgctgttccgtcaactcaa |
| ***Adpq*** | Mus Musculus | NM_009605.4 | Forward  Reverse | ggagagaaaggagatgcaggt  ctttcctgccaggggttc |
| ***Adrb3*** | Mus Musculus | NM_001768.6 | Forward  Reverse | ccttccgtcgtcttctgtgt  ttcatagccatcaaacctgttg |
| ***aP2 (Fabp4)*** | Mus Musculus | NM_024406.2 | Forward  Reverse | acaccgagatttccttcaaactg  ccatctagggttatgatgctcttca |
| ***Arbp (36B4)*** | Mus Musculus | NM_007475.5 | Forward  Reverse | tctggagggtgtccgcaac  cttgaccttttcagtaagtgg |
| ***Ccl2 (Mcp1)*** | Mus Musculus | NM_011333.3 | Forward  Reverse | caggtccctgtcatgcttct  gtggggcgttaactgcat |
| ***Ccl5*** | Mus Musculus | NM_013653.3 | Forward  Reverse | tgcagaggactctgagacagc  gagtggtgtccgagccata |
| ***Cd36*** | Mus Musculus | NM_001159558.1 | Forward  Reverse | ttgtacctatactgtggctaaatgaga  cttgtgttttgaacatttctgctt |
| ***cEbpa*** | Mus Musculus | NM_007678.3 | Forward  Reverse | caagaacaacgagtaccg  gtcactggtcaactccagcac |
| ***cEbpb*** | Mus Musculus | NM_009883 | Forward  Reverse | tgatgcaatccggatcaa  cacgtgtgttgcgtcagtc |
| ***Chi3l3*** | Mus Musculus | NM_009892.2 | Forward  Reverse | gaacactgagctaaaaactctcctg  gagaccatggcactgaacg |
| ***Cidea*** | Mus Musculus | NM_007702.2 | Forward  Reverse | ggaaaagggacagaaatggac  tcagcctgtataggtcgaagg |
| ***Cox4i1*** | Mus Musculus | NM_009941.2 | Forward  Reverse | tcactgcgctcgttctgat  cgatcgaaagtatgagggatg |
| ***Cox8b*** | Mus Musculus | NM_007751.3 | Forward  Reverse | ccagccaaaactcccactt  gaaccatgaagccaacgac |
| ***Cpt1a*** | Mus Musculus | NM_013495 | Forward  Reverse | gaagaagttcatccgattcaaga  acacccaccaccacgataag |
| ***Cpt1b*** | Mus Musculus | NM_009948.2 | Forward  Reverse | catcccaggcaaagagaca  aagcgacctttgtggtagaca |
| ***Cxcl10*** | Mus Musculus | NM_021274.1 | Forward  Reverse | ccaagtgctgccgtcattttc  ggctcgcagggatgatttcaa |
| ***Dio2*** | Mus Musculus | NM_010050.2 | Forward  Reverse | cagtgtggtgcacgtctccaatc  tgaaccaaagttgaccaccag |
| ***Elovl3*** | Mus Musculus | NM_007703.2 | Forward  Reverse | acttcgagacgtttcaggactta  gacgaccactatgagaaatgagc |
| ***Emr1*** | Mus Musculus | NM_010130.4 | Forward  Reverse | ccttggctatgggcttccagtc  gcaaggaggacagagtttatcgtg |
| ***Fasn*** | Mus Musculus | NM_007988.3 | Forward  Reverse | ccgtcacttccagttagagca  cgggtgaggacgtttacaa |
| ***Fndc5*** | Mus Musculus | NM_027402.3 | Forward  Reverse | ttgtggtcctcttcatgtgg  ttattgggctcgttgtcctt |
| ***Gapdh*** | Mus Musculus | NM_001289726.1 | Forward  Reverse | aactttggcattgtggaagg  attgggggtaggaacac |
| ***Glut4*** | Mus Musculus | NM_009204 | Forward  Reverse | gtgactggaacactggtccta  ccagccacgttgcattgtag |
| ***Hoxc9*** | Mus Musculus | NM_008272.3 | Forward  Reverse | gcagcaagcacaaagaggagaag  gcgtctggtacttggtgtaggg |
| ***Il15*** | Mus Musculus | NM_008357.1 | Forward  Reverse | cccatgtcagcagataacca  gagctggctatggcgatg |
| ***IL15ra*** | Mus Musculus | NM_008358.1 | Forward  Reverse | ccagtgccaacagtagtgaca  ttgggagagaaagcttctgg |
| ***Il1rn*** | Mus Musculus | NM_031167.5 | Forward  Reverse | tgtgccaagtctggagatga  ttctttgttcttgctcagatcagt |
| ***Il4r*** | Mus Musculus | NM_001008700.3 | Forward  Reverse | gagtggagtcctagcatcacg  cagtggaaggcgctgtatc |
| ***Il6*** | Mus Musculus | NM_031168.1 | Forward  Reverse | gctaccaaactggatataatcagga  ccaggtagctatggtactccagaa |
| ***Lipe (Hsl)*** | Mus Musculus | NM_010719.5 | Forward  Reverse | tgctcttcttcgagggtgat  gatggcaggtgtgaactgg |
| ***Lpl*** | Mus Musculus | NM_008509.2 | Forward  Reverse | ctggtgggaaatgatgtgg  tggacgttgtctagggggta |
| ***Mgl2*** | Mus Musculus | NM_145137.2 | Forward  Reverse | ggagtctccaaagtttgctctaa  aggtgggtccaagagaggat |
| ***Nos2*** | Mus Musculus | NM_010927.3 | Forward  Reverse | agcacaggaaatgtttcagc  atcagcttgcaagaccagag |
| ***Nrf1*** | Mus Musculus | NM_001164226.1 | Forward  Reverse | gacgctgctttcagtccttc  gtgttcagtttgggtcactcc |
| ***Ppara*** | Mus Musculus | NM_011145.3 | Forward  Reverse | cgggaacaagacgttgtcat  cagataagggactttccaggtc |
| ***Ppard2*** | Mus Musculus | NM_011145.3 | Forward  Reverse | agaacacacgcttccttcca  ccgacattccatgttgagg |
| ***Pparg*** | Mus Musculus | NM_011146.3 | Forward  Reverse | tgctgttatgggtgaaactctg  ctgtgtcaaccatggtaatttctt |
| ***Prdm16*** | Mus Musculus | NM_001177995.1 | Forward  Reverse | aaggggaaggagagatacacg  cagatgtcttgtgagatttgcag |
| ***rpL19*** | Mus Musculus | NM_009078.2 | Forward  Reverse | ccacaagctctttcctttcg  ggatccaaccagaccttcttt |
| ***Sfrp5*** | Mus Musculus | NM_018780.2 | Forward  Reverse | gaaagttgattggagcccagaa  gcccgtcaggttgtctaactgt |
| ***Tbp*** | Mus Musculus | NM_013684.3 | Forward  Reverse | cggtcgcgtcattttctc  gggttatcttcacacaccatga |
| ***Tfam*** | Mus Musculus | NM_009360.4 | Forward  Reverse | caaaggatgattcggctcag  aagctgaatatatgcctgcttttc |
| ***Tlr2*** | Mus Musculus | NM_011905.3 | Forward  Reverse | ggggcttcacttctctgctt  agcatcctctgagatttgacg |
| ***Tlr4*** | Mus Musculus | NM_021297.2 | Forward  Reverse | ggactctgatcatggcactg  ctgatccatgcattggtaggt |
| ***Tnfa*** | Mus Musculus | NM_013693.2 | Forward  Reverse | cgtcgtagcaaaccaccaag  gagatagcaaatcggctgacg |
| ***Ucp1*** | Mus Musculus | NM_009463.3 | Forward  Reverse | cctgcctctctcggaaacaa  tgtaggctgcccaatgaaca |
| ***Ucp2*** | Mus Musculus | NM_011671.4 | Forward  Reverse | acagccttctgcactcctg  ggctgggagacgaaacact |
| ***Ucp3*** | Mus Musculus | [NM_009464.3](https://qpcr.probefinder.com/showsequence.jsp?seqNo=672573315) | Forward  Reverse | ggatgcctacagaaccatcg  ttgtgatgttgggccaagt |
|  |  |  |  |  |
| ***CCL2*** | homo sapiens | NM_002982.3 | Forward  Reverse | catagcagccaccttcattc  ggtcagcacagatctccttg |
| ***CD36*** | homo sapiens | NM_001289911.1 | Forward  Reverse | agttctcaatctggctgtgg  cggaaccaaactcaaaaatg |
| ***CD4*** | homo sapiens | NM_000616.4 | Forward  Reverse | tcaaaatagacatcgtggtgct  acctgttccccctctttctta |
| ***CD68*** | homo sapiens | NM_001251.2 | Forward  Reverse | agcccagattcagattcgag  tggttttgttggggttcagt |
| ***CD8*** | homo sapiens | NM_001768.6 | Forward  Reverse | tcatggccttaccagtgacc  aggttccaggtccgatcc |
| ***HOXC9*** | homo sapiens | NM_006897.1 | Forward  Reverse | cagcaagcacaaagaggaga  cgacggtccctggttaaatac |
| ***IL15*** | homo sapiens | NM_172175.2 | Forward  Reverse | caaacaacagtttgtcttctaatgg  gacaatatgtacaaaactctgcaaaaa |
| ***IL15RA*** | homo sapiens | NM_001243539.1 | Forward  Reverse | ttgccttgacttgaggtagtagc  agacaacagccaagaactgg |
| ***IL6*** | homo sapiens | NM_000600.3 | Forward  Reverse | gtgtgaaagcagcaaagaggc  ctggaggtactctaggtatac |
| ***PGC1A*** | homo sapiens | NM_013261.3 | Forward  Reverse | accatattccaggtcaagatcaa  gcttgactcatagtaatagcaggatct |
| ***PPARG*** | homo sapiens | NM_138712.3 | Forward  Reverse | agtcctcacagctgtttgccaagc  gagcgggtgaagactcatgtctgt |
| ***PRDM16*** | homo sapiens | NM_022114.3 | Forward  Reverse | gaaactttattgccaatagtgagatga  ccgtccacgatctgcatgt |
| ***UCP1*** | homo sapiens | NM_021833.4 | Forward  Reverse | tccccggtggatgtagtaaa  cgcaagaaggaaggtaccaa |
